# Physical activity and nutrition interventions for older adults with cancer: a systematic review

**DOI:** 10.1007/s11764-020-00883-x

**Published:** 2020-04-24

**Authors:** Cynthia C. Forbes, Flavia Swan, Sarah L. Greenley, Michael Lind, Miriam J. Johnson

**Affiliations:** 1 Wolfson Palliative Care Research Centre, Hull York Medical School, University of Hull, Kingston-Upon-Hull, UK; 2 Present Address: University of Hull, Allam Medical Building 3rd Floor, Cottingham Road, Kingston-Upon-Hull, East Yorkshire HU6 7RX UK; 3 Academy of Primary Care, Hull York Medical School, University of Hull, Kingston-Upon-Hull, UK; 4 Cancer Research Group, Hull York Medical School, University of Hull, Kingston-Upon-Hull, UK

**Keywords:** Neoplasms, Rehabilitation, Diet, Exercise, Well-being, Quality of life, Review, Aged, Health behaviour

## Abstract

**Purpose:**

The aim of this review was to summarize the current literature for the effectiveness of activity and nutritional based interventions on health-related quality of life (HRQoL) in older adults living with and beyond cancer (LWBC).

**Methods:**

We conducted systematic structured searches of CINAHL, Embase, Medline, Cochrane CENTRAL databases, and bibliographic review. Two independent researchers selected against inclusion criteria: (1) lifestyle nutrition and/or activity intervention for people with any cancer diagnosis, (2) measured HRQoL, (3) all participants over 60 years of age and (4) randomized controlled trials.

**Results:**

Searches identified 5179 titles; 114 articles had full text review, with 14 studies (participant *n* = 1660) included. Three had nutrition and activity components, one, nutrition only and ten, activity only. Duration ranged from 7 days to 1 year. Interventions varied from intensive daily prehabilitation to home-based gardening interventions. Studies investigated various HRQoL outcomes including fatigue, general and cancer-specific quality of life (QoL), distress, depression, global side-effect burden and physical functioning. Eight studies reported significant intervention improvements in one or more QoL measure. Seven studies reported using a psychosocial/theoretical framework. There is a gap in tailored nutrition advice.

**Conclusions:**

Among the few studies that targeted older adults with cancer, most were activity-based programmes with half reporting improvements in QoL. Future research should focus on or include tailored nutrition components and consider appropriate behaviour change techniques to maximize potential QoL improvement.

**Implications for Cancer Survivors:**

More research is needed to address the research gap regarding older adults as current recommendations are derived from younger populations.

**Electronic supplementary material:**

The online version of this article (10.1007/s11764-020-00883-x) contains supplementary material, which is available to authorized users.

## Background

The proportion of adults aged 65 or older in the United Kingdom (UK) was estimated to be about 18% in 2017, with projections of an increase to around 24% by 2037 [1]. Just under two-thirds of new cancer cases in the UK, on average each year are in people aged 65 and over [2–5]. Many people post-diagnosis live with multiple adverse side effects that impact both physical and mental health. Cancer treatments are also associated with higher rates of other conditions like cardiovascular disease, type 2 diabetes and subsequent primary cancers [6].

In addition, 1 in 10 people aged 65 years or older is affected by frailty [7]. Frailty is a clinical syndrome characterized by multisystem decline that leads to lower functional reserve, increased vulnerability to dependency and mortality after minor stressor events [8]. Frailty is also associated with adverse outcomes such as increased risk of falls, disability, hospitalization and death [9]. Older adults with cancer are at higher risk of frailty than their younger counterparts. This may limit chemotherapy and other therapeutic options or result in dose reductions and low treatment completion rates.

When coupled with higher rates of sarcopenia (the progressive degeneration of skeletal muscle mass), cachexia (extreme weight loss and muscle wasting due to chronic illness) and nutritional deficiencies (e.g. malnutrition, etc.), cancer and its treatment confer a range of effects which reduce quality of life (QoL) [10]. One recent study found that nearly two-thirds of older people assessed in hospital had at least one tissue loss syndrome (i.e. sarcopenia, frailty, cachexia or malnutrition) [11]. This is concerning as sarcopenia, for example, has been independently associated with 1-year mortality rates in older adults with cancer [12]. Obesity and fat gain have also been identified as a health issue that will become more common among older adults LWBC as the proportion of the general population classified as overweight and obese continues to increase [13, 14]. The American Society of Clinical Oncology (ASCO) has even urged clinicians to intervene and counsel patients, agreeing that obesity is a major concern among people LWBC [15, 16].

Physical activity (PA) benefits people living with or beyond cancer by improving physical function and QoL during and after cancer treatment, and cancer-related outcomes like treatment completion, maintenance of, or faster return to, pre-treatment health, fewer unnecessary healthcare visits and better survival rates [6, 17–22]. Improvements are greater in those engaging in PA sooner after a diagnosis [23]. Rehabilitation among people with chronic obstructive pulmonary disease (COPD) is also known to reduce improve function short term [24]. Emerging work indicates that exercise and immune function in the older person are related [25–30].

Poor nutritional status is associated with worse overall survival and QoL in patients receiving chemotherapy than those with better nutritional status [31, 32]. A recent review suggests that nutritional interventions, including dietary counselling and a multi-modal approach of exercise and nutrition, may support well-being and patient’s ability to complete treatments; however, further high-quality research is needed [33].

A tailored activity and nutrition intervention, designed to optimize physical function and nutritional status irrespective of treatment plan, started soon after diagnosis may increase the percentage of older people able to complete chemotherapy, and improve QoL and functional ability in those unfit for chemotherapy. Previous work has focused on prehabilitation (e.g. prior to surgery) [34, 35], maintenance during treatment (e.g. alongside chemotherapy) [36] or rehabilitation for cancer survivors post-treatments [37, 38].

Older adults are a growing proportion of the general and cancer populations; yet, they are underrepresented in clinical trials [39, 40]. In fact, a systematic review found that of all RCTs assessed in a 1-year period, only 3% were specifically designed for adults age 65 or older [41]. Additionally, older adults are often excluded based on secondary cancers, co-morbidities and declines in physical function and cognition [42]. The majority of guidance for lifestyle behaviour change in cancer has been derived from early stage breast and prostate cancer populations, a generally younger, fitter, group [43]. As such, recommendations may not be appropriately generalized to older groups of poorer health, for example, adults with lung cancer, the proportion of which being aged 65 or older is 78% [2–5].

The benefits of exercise in the non-cancer population have widespread acceptance and an extensive evidence base [43], but previous research relating to exercise in cancer patients is less robust and has not been tailored to the older or frail adult. Conversely, programmes developed for older adults have not included people with cancer. The Cancer and Ageing Research Group in Wisconsin observed that “simply extracting results from the larger body of geriatric exercise trials is not sufficient to inform how exercise is prescribed for geriatric oncology patients” [44]. They recommend careful work regarding patient population selection, development of the intervention and choice of outcome measurement to enable rigorous development and testing of programmes prior to rollout in clinical practice. Therefore, we aimed to summarize the current literature regarding activity and nutritional based interventions on health-related quality of life (HRQoL) in older adults with cancer delivered before, during or after active cancer treatments, or as part of best supportive care.

## Methods

### Study design

The conduct and reporting of this review adhere to the Preferred Reporting Items for Systematic Reviews and Meta-analyses (PRISMA) [45]. A data charting/extraction form was adapted from the Johanna Briggs Institute (JBI) Reviewers’ Manual: Methodology for JBI reviews (2015) [46]. A copy of the final form can be found on our open science framework page (https://osf.io/p23jd/).

### Inclusion and exclusion criteria

Studies were included if they met the following a priori eligibility criteria: (1) delivered a lifestyle intervention for nutrition and/or PA to people with any cancer diagnosis, (2) included a measure of HRQoL, (3) participants over 60 years or at least 50% over 60 years with data analyses by age group and (4) randomized controlled trials. Studies were excluded if (1) we could not determine an age range, (2) the intervention was targeting clinicians or carers rather than older adults with cancer, (3) publication language was not in English or (4) findings were conference abstracts only.

### Search strategy

Studies were identified through structured searches of all publication years (final update search performed 30 May 2019) in the following electronic databases: Medline via OVID, Embase via OVID, Cochrane Central Register of Controlled Trials (CENTRAL) and Cinahl via EBSCO. The search strategy was developed in consultation with a specialist librarian at the University of Hull and finalized with the aid of an information specialist. MeSH terms in Medline (see supplemental file 1) were developed to search for all key concepts and modified for other databases. Keyword searches restricted to abstract and title were also completed. Boolean logic was used to combine the terms. The original database searches were conducted by a single author (CF) and updated by an information specialist (SG). For the updated search, search filters for RCTs including Cochrane’s Highly Sensitive Search Strategy were included to retrieve randomized controlled trials.

### Study selection

All identified articles were uploaded into an EndNote X8 database and duplicates removed. Preliminary screening was undertaken by one author (CF) to remove obvious exclusions (e.g. conference abstracts, etc.) after which two authors (CF and SG) independently screened all articles against eligibility criteria taking title, abstract and full-text into account. Disagreements were discussed and resolved by consensus. Any unresolved items were reviewed by a third author (FS) and their decision stood. If criteria were unclear in the manuscript, corresponding authors were emailed and asked for clarification.

### Data extraction

A data extraction form was developed and piloted by the research team to extract data about study details and characteristics (e.g. country, setting, sample characteristics, etc.), intervention details (e.g. group descriptions, intervention components and duration, etc.), QoL outcomes and key findings and messages. The form was independently tested using one article by two authors (CF and SG) and revised following discussion. Data were then extracted using the form by a single reviewer (CF). A second (SG) and third (FS) reviewer randomly selected two articles each (i.e. 25%) and reviewed the data extracted. As there were no discrepancies, data extraction by a second reviewer for the remaining articles was considered unnecessary.

### Risk of bias assessment

Two authors (CF and SG) used the Risk of Bias Assessment Tool version 2 available from the Cochrane handbook (2011) to independently assess quality of life outcomes from all included studies. The articles were judged for bias as either low, high or some concerns for the following: (1) selection (random sequence generation and allocation concealment), (2) performance (blinding of participants and study personnel), (3) detection (blinding of outcome assessment), (4) missing outcome data and (5) reporting (selective outcome reporting). The nature of lifestyle behaviour change studies means double-blinding is very difficult but this tool allows fair judgements despite this fact. The authors discussed any differences and reached consensus; therefore, a third party was not necessary.

### Outcomes

To describe the nature of studies currently targeting older adults with cancer, we extracted detailed information related to the intervention groups including (1) type of intervention, (2) intervention delivery methods, (3) all components of intervention, (4) study duration and measurement timing and (5) comparator group information. The primary efficacy outcomes of interest for this review were measures related to QoL or HRQoL. The primary outcomes for each study were identified and noted.

## Results

### Study selection

**Fig. 1:**
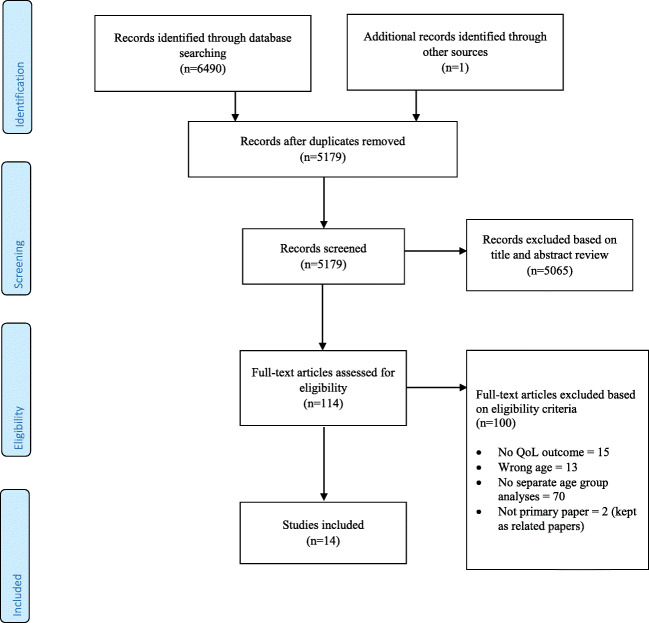
PRISMA flow diagram

The study selection process is presented in Fig. 1. A total of 6490 records were identified; 5179 remained after de-duplication. After title and abstract screening, 114 articles were identified for full-text review. Of those, 14 studies were deemed eligible and were included in full data extraction for this review.

### Study characteristics

This review included 14 papers [47–60]. A further eight related papers were referenced to provide more detail when unable to find information in the primary paper (e.g. protocol papers) [61–68]. One study was a three-arm randomized controlled trial (RCT) [47] with the remaining 13 studies being two-arm RCTs. One study was described as a pilot [51] and seven as feasibility [47, 48, 50, 54, 55, 58, 60] RCTs. Seven studies took place in the USA [49, 50, 55, 56, 59, 60], two in Canada [47, 51] and one each in China [52], Japan [54], Korea [57], Sweden [58] and UK [48]. Five studies recruited patients with prostate cancer only [47, 48, 55, 57, 60], one each recruited bladder [58], lung [52] and breast cancer only [51], with the remaining recruiting a mixed sample of cancer types [49, 50, 53, 54, 56, 59].

### Risk of bias assessment

**Fig. 2:**
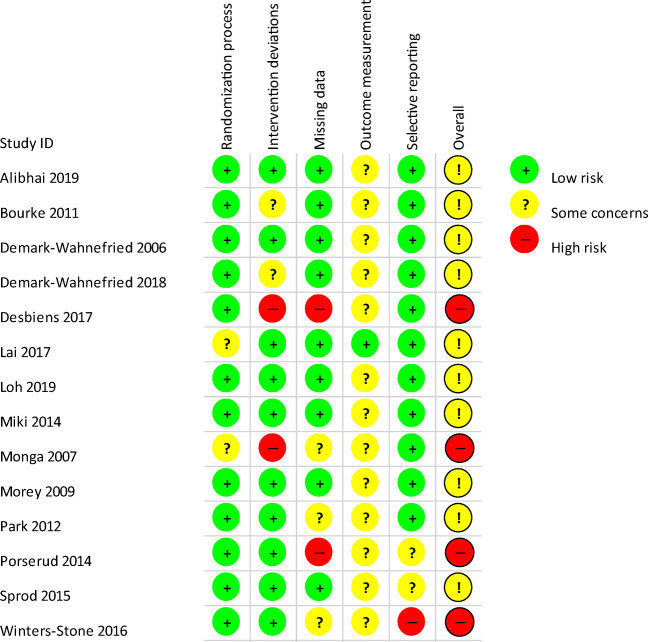
Risk of bias summary diagram

**Fig. 3:**
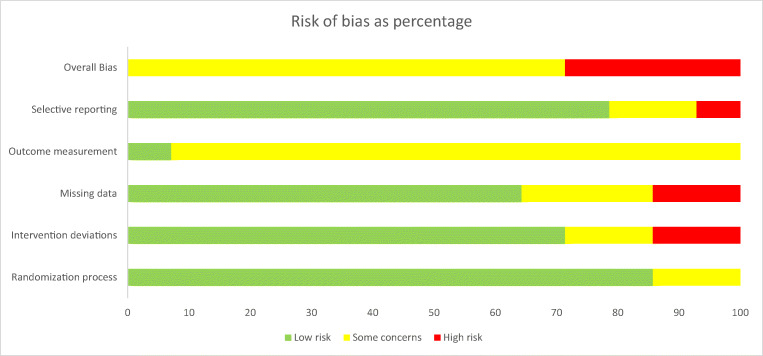
Risk of bias represented as a chart showing percentages

Full results from the assessment can be found in Figs. 2 and 3. Based on assessments from two reviewers, no studies received overall risk of bias judgements of low, ten were judged to have some concerns and four had high risk of bias. Twelve studies were low [47–51, 53, 54, 56–60] and two some concerns [52, 55] for allocation; ten were low [47, 49, 52–54, 56–60], two some concerns [48, 50] and two high [51, 55] for intervention deviations; nine rated low [47–50, 52–54, 56, 59], three some concerns [55, 57, 60] and two high [51, 58] for missing data; one ranked low [52] and 13 some concerns [47–51, 53–60] for outcome measurement; and finally, 11 rated low [47–57], two some concerns [58, 59] and one high [60] for selective reporting.

### Intervention characteristics

Most interventions focused on PA behaviour only [47, 51–55, 57–60] and three on both nutrition and activity behaviour [48, 49, 56]. One study reported improving nutrition as its main objective; however, this was a gardening intervention which could also be considered activity [50]. The studies ranged in duration from 7 days to 1 year, six studies [48–50, 56, 58] collected follow-up measures beyond post-study though only three studies reported this data in the included articles [48, 49, 58]. Intervention settings included hospital [52–55, 58], community [57, 59, 60], home [49, 50, 56] or a mixture of settings [47, 48, 51]. Detailed characteristics of the included studies can be found in Table 1.

**Table 1:** Characteristics of studies included in review

Source	Design	Setting	Sample	Timing of intervention	Intervention focus	Comparator	Follow-up
Alibhai 2019 [47]	3-arm feasibility RCT	Canada Hospital or home	59 men with prostate cancer mean age 70 (range 62–90)	Starting or continuing ADT	Activity	Non-inferiority comparison between home-based activity (HOME; intervention group), group-based face-to-face (GROUP), and 1:1 personal training (PT).	3- and 6-month follow-ups (not reported in this paper)
Bourke 2011 [48]	2-arm feasibility RCT	UK Home or supervised (unknown whether hospital or community)	50 men with localised prostate cancer mean age 72 (range 60–87)	receiving AST for at least 6 months	Nutrition and activity	Usual care	6 months
Demark-Wahnefried 2006 [49]	2-arm RCT	USA Home	182 breast and prostate cancer patients Mean age 72 (range 65–91)	Within 18 months of diagnosis	Nutrition and activity	Attention control Received workbook with general health promotion materials. Also received phone counselling (same schedule) that was structured around a specific health topic as addressed in workbook.	12 months
Demark-Wahnefried 2018 [50]	2-arm feasibility RCT	USA Home	46 mixed cancer survivors Mean age 70 (range 60–92)	Post treatment (exception for adjuvant endocrine therapy)	Nutrition	Wait list control Matched with MGs, given all materials and monitored for year 2 after 1 year wait-list.	24 months
Desbiens 2017 [51]	2-arm pilot RCT	Canada Home or hospital based	26 breast cancer currently on aromatase inhibitor therapy	No RT for at least 1 month	Activity	Active comparator Same activity routine in group setting.	None
Lai 2017 [52]	2-arm RCT	China Hospital	60 lung cancer patients	Pre-surgery	Activity	Usual care	None
Loh 2019 [53]	2-arm RCT	USA Hospital	252 mixed cancer patients	currently or soon be receiving chemotherapy	Activity	Wait list control Given the intervention kit for free at the end of the study	None
Miki 2014 [54]	2-arm feasibility RCT	Japan Hospital	78 breast (43) and prostate (35) cancer	Majority post treatment 5 chemo, 2 radiation, 41 hormone	Activity	Usual care	None
Monga 2007 [55]	2-arm feasibility RCT	USA Hospital	21 localised prostate cancer patients	Receiving radiotherapy	Activity	Usual care plus general education	None
Morey 2009 [56]	2-arm RCT	USA (small number from UK and Canada) Home	641 breast, prostate, and colorectal cancer survivors randomised Mean age 73 (no range)	5 or more years post diagnosis (considered to be cured)	Nutrition and Activity	Wait list control Usual care until 1 year when they received the full intervention.	24 months
Park 2012 [57]	2-arm RCT	Korea University	66 localised prostate cancer patients randomised Mean age 69 (no range)	post prostatectomy; excluded if any adjuvant or neoadjuvant therapy	Activity	Usual care plus kegel exercises only.	None
Porserud 2014 [58]	2-arm feasibility RCT	Sweden University hospital	18 bladder cancer patients randomised Mean age 72 (range 64–78)	After undergoing radical cystectomy for bladder cancer	Activity	Wait list control Usual care during study period offered same programme after data collection was complete.	12 months
Sprod 2015 [59]	2-arm RCT	USA Community, Cancer centre	97 older (≥ 60) mixed cancer survivors randomised Mean age 66 (no range)	completed standard treatment 2–24 months prior	Activity	Wait list control Offered opportunity to participate in sessions post study.	None
Winters-Stone 2016 [60]	2-arm feasibility RCT	USA University	64 prostate cancer survivors and their spouse or partner (64 couples) randomised Mean age of PCS 72; mean age of spouses 68	received treatment for PCS but not currently on CT or RT	Activity	Wait list control Received home-based instructional video of the programme and an instructional workshop post-study	None

*QoL* quality of life, *RCT* randomised controlled trial, *UK* United Kingdom, *USA* United States of America, *ADT* androgen deprivation therapy, *AST* androgen suppression therapy, *PT* personal training, *MG* master gardener, *PCS* prostate cancer survivors, *CT* chemotherapy, *RT* radiotherapy

### Activity intervention characteristics

Eight studies included some specific form of aerobic activity [47, 48, 51, 52, 54–56, 58], five had traditional-style strength training (e.g. lifting weights or using resistance bands) [47, 48, 56, 58, 60] and five included specific training to build balance and/or functional strength [51, 52, 57–59]. Seven interventions included at least one supervised individual session [47, 52–55, 57, 60], seven had home-based activities [47–51, 53, 56] and four were group-based classes or training sessions [47, 48, 58, 59]. Home-based interventions were delivered using DVD [51], booklets/binders [48–50, 53, 56] and four included visits or telephone calls to check-in and determine progress [47, 49, 50, 56]. Six home-based studies included personalized advice on activity [47–49, 51, 55, 56].

### Nutrition intervention characteristics

One study provided participants with all supplies and guidance on growing their own vegetables at home and had role models in the form of a Master Gardener to teach and assist [50]. One other study provided portion-control tableware to assist eating habits and included tailored nutrition advice as compared to national guidelines [56]. One study had personalized comparisons to general information regarding standard nutrition guidelines [49]. One study had a series of “healthy eating” seminars and a nutrition advice pack [48].

### Theoretical intervention characteristics

Three studies explicitly stated they used a theory to develop study materials including social cognitive theory [49, 50, 56], transtheoretical model [49] and social ecological model [50]. Other studies included information regarding habit formation [48], autonomy [48], self-efficacy [49] and action/coping planning [47, 50]. Aside from those studies with group-based session, to increase social support, one study formally included spouses or partners in the intervention [60] and one had a private Facebook group for study participants [50]. Detailed descriptions of the interventions included can be found in Table 2.

**Table 2:** Study intervention components

Source	Intervention description	Delivered by	Mode of delivery	Location of intervention activities	Intervention length, frequency, duration, intensity	Level of tailoring; how was this accomplished?
Alibhai 2019 [47]	Participants were an exercise programme of mixed modality exercise incorporating aerobic, strength and flexibility training. All training programmes followed the FITT principle. An education component was included and focused on common concerns facing new exercisers. This occurred during sessions or phone calls throughout the intervention period. All participants received resistance bands for home-based sessions. HOME group also received a stability ball, exercise mat, HR monitor with instructions, and a smartphone with a 6-month paid talk and data plan for phone check-ins.	A certified exercise physiologist delivered instructions and an orientation of exercises to all participants. CEP delivered PT and GROUP sessions.	Initial session was face to face for all participants. PT group received 1:1 face to face sessions. GROUP received supervised sessions in groups of 4–6 individuals HOME had weekly phone calls. All participants received a print-based instruction manual to supplement home-based sessions.	PT and GROUP sessions were described as “in-centre” and they were encouraged to do additional home-based sessions as the intervention progressed. HOME intervention activities were all home-based.	Intervention period was 6 months. Relative intensity was maintained throughout the programme based on baseline measures ensuring similar progression between the groups. Each session consisted of cardiovascular training for 15–30 min, strength training (working major muscle groups), and flexibility training (including 5–10 min of stretching at the end of each session). PT and GROUP had 3 in-centre sessions per week for 6 months. Participants were asked to do 4–5 sessions in total per week.	Programmes were tailored based on baseline fitness assessments with target HR set at 60–70% of HRR.
		Health coaches delivered weekly phone calls to HOME group.				
Bourke 2011 [48]	*Activity component* Participants were provided with an exercise programme consisting of aerobic and strength training. Supervised sessions were intended to provide education on correct exercise performance and technique, and guidance on heart rate and RPE Home-based sessions were self-directed PA of their choosing. A log book was used to keep track. *Nutrition component* Participants were given a nutrition advice pack that encouraged: reducing saturated fat and refined carbs, increasing fibre, moderation of alcohol Diet information given as “advice” purposefully so to allow choices to be made by participant.	Supervised sessions delivered by ‘an experience exercise physiologist’. Unknown who delivered healthy eating seminars.	Supervised sessions were face to face Healthy eating seminars were in a small-group setting	Unclear if supervised sessions were in hospital or a community setting but were in a “dedicated exercise suite”; remainder were home-based sessions Unclear if healthy eating seminars were in hospital or a community setting	The intervention was 12 weeks in length. Minimum 3 sessions per week (weeks 1–6: 2 supervised, 1 home; weeks 7–12: 1 supervised; 2 home) but patients were encouraged to get PA 5 days per week. Supervised exercise sessions: 30 min of aerobic PA at 55–85% age predicted max HR and RPE of 11–15, followed by strength training comprised of 2–4 sets targeting large muscle groups. Home-based sessions were 30 min in length. Healthy Eating seminars were 15–20 min in length, held fortnightly throughout the 12 weeks	Baseline testing assessing physical function was used to determine appropriate starting points for aerobic and strength training intensities.
						A behavioural component of the supervised sessions included exploring, with each participant, how to make PA a habit in daily life, identifying and using available social support, preferred types of PA.
Demark-Wahnefried 2006 [49]	Participants received a personally tailored workbook with diet and exercise information based on their current stage of readiness to change. Periodic telephone counselling from qualified experts accompanied the workbook and were intended to help participants develop a plan to achieve goals, answer questions, guide them through workbook, and monitor progress. Participants were given a pedometer and log book for self-monitoring. Diet and PA feedback was provided in the workbooks based on their self-reported intake and compared to national guidelines for total fat, saturated fat, cholesterol, vegetables, fruits, whole grains, calcium, iron, and current PA levels. Also received standardised materials regarding dietary components and “Exercise: a guide from the National Institute of Aging”	First 3 months of counselling sessions (focusing on diet) were delivered by a registered dietician.	Study materials were delivered to participants via post. Counselling sessions were delivered via telephone. Microsoft Access forms were developed to standardise and guide counsellors through sessions while collecting process data concurrently.	Intervention activities were distance-based.	Intervention was 6 months. Tailored workbooks were mailed at the beginning of the study period. Telephone counselling sessions were up to 30 min in length, fortnightly for the 6 month study period.	Both dietary and PA feedback in the workbooks were tailored to self-reported data on baseline measures. For each participant, the top 3 sources of dietary fat, saturated fat, and cholesterol were identified and tips for improving intake was included. Telephone counselling was tailored to specific nutritional deficiencies and/or functional limitations noted from the baseline survey. tailored using stages of change from the TTM and SCT to increase likelihood of behavioural change.
		Final 3 months of counselling sessions (focusing on PA) were delivered by an exercise physiologist.				
Demark-Wahnefried 2018 [50]	Participants were given either a raised garden bed or 4 EarthBoxes (good for apartments, townhomes, low light areas etc.) and gardening supplies for a spring, summer, and fall garden. They were able to keep all the supplies. Master Gardeners were match with participants based on geographic proximity and introduced at a meet n’ greet event. MGs worked with their assigned people to plan, plant, tend, and harvest three gardens over the course of a year. Each participant received a notebook with general information on gardening, cancer-specific concerns, and contact information for MG and study personnel.	Gardening advice and assistance provided by Master Gardeners who had undergone 100 h of training through the Alabama Cooperative Extension System. MGs were trained to promote self-efficacy by being role models, encouraging goal setting, giving reinforcement and encouragement, strategizing to overcome barriers, and skills training.	Intervention was delivered via face-to-face home visits, printed materials, and telephone calls. Participants were encouraged to participate in an online Facebook group as a form of social support.	Intervention activities were home-based.	Intervention length was 12 months.	Only tailored in the types of plants grown as participants were able to plan their preferred garden. Overall intervention was guided by SCT and SEM.
					MGs checked in fortnightly alternating between phone or email check-ins and home visits.	
Desbiens 2017 [51]	This study compared two methods of delivering the same activity programme. One group performed individual, home-based exercise with the assistance of videos; another group performed the same activities in a group-based setting. Principles of activity training that were used to develop the programme are as follows: 1) specificity; 2) progression; 3) overload; 4) initial values; 5) reversibility; and 6) diminishing returns.	Exercise programme was developed and delivered by a kinesiologist. Exercises were approved by a surgical oncologist. Videos were produced by researchers featuring a kinesiologist performing activities at three different intensities. Group-based had the same exercises delivered face-to-face by the same kinesiologist.	This study compared individual video-assisted, home-based activity versus group-based activity.	Video-assisted group had intervention as home-based. Unclear where group-based activities were held.	Intervention was 12 weeks in length. Participants were asked to perform programme minimum twice per week for 12 weeks. Exercise routine was 50 min in total: 5 min warm up; 15 min cardiovascular exercise; 20 min muscle reinforcement; 10 min relaxation. Three levels of intensity were proposed to each participant and they selected the preferred level based on their own energy levels.	No tailoring
Lai 2017 [52]	Elderly participants awaiting lobectomy were provided a prehabilitation programme that focused on improving lung fitness and cardiopulmonary intolerance to subsequently reduce postoperative pulmonary complications.	Participants were “assessed and data were recorded” by a physiotherapist, but it is unclear whether they also delivered the intervention or whether it was delivered by a member of the study team.	Intervention was delivered face-to-face.	Activity training took place in the rehabilitation centre within the hospital.	The intervention was 7 days in length. Daily activity training consisted of: 1) abdominal breathing training 2 times per day for 15–20 min, 2) expiration exercises with Voldyne 5000 3 times per day for 20 min; 3) 30 min of aerobic endurance training on Nustep device (at the speed and power of their choice).	No tailoring
Loh 2019 [53]	The intervention was a home-based, low-to moderate-intensity walking and strength training programme. Participants in the intervention arm were given an exercise kit, containing a pedometer, three resistance bands (medium, heavy, and extra heavy intensity), and an instruction manual. Aerobic component was an individually tailored, progressive walking programme based on baseline number of steps. Strength training was performed with therapeutic resistance bands.	A designated clinical research associate was trained by an American College of Sports Medicine–certified exercise physiologist from the URCC Research Base to teach the programme to participants	Education session was face-to-face. Exercise sessions were delivered via print materials.	Intervention was home-based delivery	The intervention was 6 weeks long Participants recorded their steps daily and were encouraged to progressively increase their steps by 5% to 20% every week. Participants were asked to perform 10 required exercises (e.g. squat or chest press) and four optional exercises daily following an individually tailored set/repetition scheme. They were encouraged to progressively increase intensity, sets, and/or number of repetitions over course of the programme.	Participants were prescribed an individually tailored walking programme based on a 4-day pedometer measurement at baseline. Unclear how the strength training component was tailored.
Miki 2014 [54]	Speed feedback therapy with bicycle ergometer connected to computer was conducted. Participants pedalled to match the arbitrary speed displayed on the computer screen. Pedalled while visually tracking a path and modifying their speed to follow the path.	Sessions were conducted by rehabilitation therapists.	Intervention was delivered face-to-face.	Intervention took place in the rehabilitation room within the university hospital.	The intervention was 4 weeks in length Participants completed 1 session per week for 4 weeks. Exercise load was set to 20 W and max RPMs of 80 for a pedalling time of 5 min	No tailoring
Monga 2007 [55]	Participants completed aerobic exercise sessions in the morning before receiving radiation therapy.	Programme was conducted by a staff kinesiotherapist and supervised by physician	Intervention was delivered face-to-face.	Intervention was delivered in the medical centre	The intervention was 8 weeks in length. Participants exercised 3 times per week, in the morning before their RT Sessions consisted of 10 min warm up, 30 min treadmill walking, 5–10 min cool down. Intensity of 65% HRR was the target for patients. Weekly HR measures were taken and recalculations done for target HR if necessary	Intensity was tailored to individual HR from baseline and subsequent measures.
Morey 2009 [56]	Participants were provided a personally tailored workbook that compared and gave feedback on current self-reported physical activity and diet behaviour to national guidelines. Participants received a pedometer, a set of resistance bands (3 levels of resistance), and an exercise poster with 6 lower body strength exercises targeting physical function. The nutrition component of the intervention included “Portion Doctor tableware”, a fat gram book to help monitor fat intake, a pocket magnifier, and personalised record logs.	Unclear who specifically delivered telephone counselling sessions (i.e. study team, hired staff, etc.) and how they were trained. To standardise data collection and message delivery, counsellors used computer-assisted templates with branching algorithms to guide counselling sessions.	Intervention was print materials delivered via post along with phone follow-ups. Telephone counselling sessions to help establish rapport and enhance social support.	Intervention was home-based.	The intervention period was 12 months No specific prescriptions were given but recommendations were 15 min of strength exercise every other day, 30 min of endurance exercise each day. Telephone counselling (15–30 min in length) was scheduled weekly for 3 weeks, then 2 fortnightly, then monthly for the rest of the year.	First few pages of the workbook content was tailored based on the self-reported baseline measurements. Personalised progress reports were mailed every 12 weeks consisting of 2 pages tailored for each person’s stage of readiness and comparing their self-reported behavioural change over time. Print materials and telephone scripts based primarily on SCT, operationalised the key concepts of behavioural capacity, outcome expectancies, self-control, reinforcement and self-efficacy.
Park 2012 [57]	Men after a radical prostatectomy participated in an exercise programme designed to improve exercise ability, QoL, and incontinence. Exercises were progressed over the 12 weeks. Initially focused on pelvic floor exercises (weeks 1–4), then incorporating stability ball exercises (weeks 5–8), and finally resistance band exercises (weeks 9–12). Kegel exercises were also performed.	Exercises were performed by “sports experts” Unclear specifically who delivered the intervention	Intervention was delivered face-to-face. Unclear whether supervised sessions were group-based or individual.	Unclear whether in hospital or university setting.	The intervention was 12 weeks in length. Programme was initiated during postop week 3 and was conducted for 12 weeks thereafter. Participants exercised 2 times per week, for about 60 min per day. Kegel exercise instructions were to do 3 daily sessions, 30 repetitions of a 1–5 s contraction	Intensity of exercises were tailored to HRR of each participant.
Porserud 2014 [58]	The intervention started within a week of baseline assessment with the aim to increase physical function. The programme consisted of strength and endurance training for the lower extremities like walking, strengthening exercises, balance training, mobility training, and stretching.	Led by physiotherapists	Face-to-face group sessions were held.	Sessions took place at the university hospital where participants were recruited.	Intervention was 12 weeks in length Sessions were 45 min in length, twice per week over the study period. Also instructed to take walks 3–5 days per week for at least 15 min at a self-selected pace.	Sessions were adapted for individual capabilities but otherwise not tailored.
Sprod 2015 [59]	Yoga for Cancer Survivors (YOCAS) intervention consisted of a standardised programme consisting of breathing exercises, postures, and mindfulness exercises. Breathing exercises included slow, controlled, and diaphragmatic breaths and breathing coordinated with movement. Postures included 16 gentle hatha and restorative yoga poses, of which there are seated, standing, transitional, and supine poses. Meditation exercises included meditation, visualization, and affirmation.	Instructors were all Yoga Alliance registered and received a dvd and instructions in addition to training with the PI to ensure they were all delivering the programme as described. They were not allowed to add or remove anything but could modify as necessary.	Face-to-face group sessions	Small regional cancer centres or yoga studios	The intervention lasted 4 weeks. Participants were expected to attend sessions lasting 75 min each, twice per week over 4 weeks for a total of 8 sessions. There was no option to make up missed sessions. Exercises were generally considered low intensity (< 3 METs)	No tailoring
Winters-Stone 2016 [60]	Participants and their spouses engaged in an exercise programme. Exercises were performed as trainer/ coach to promote teamwork and assist with form, motivation etc. then roles were switched. Some exercises were performed at the same time or in tandem including chair rises, 90 degree squats, lunges, 1 arm rows, bench press, push ups, triceps extensions, and shoulder raises.	All classes were instructed by the same Exercise Physiologist	Group-based face-to-face classes	Sessions took place at Oregon Health & Science University.	The intervention period was 6 months in length. Participants attended 2 sessions per week for the 6 month period with their partner. Each class was 60 min long and held with other couples. Participants could attend solo if their spouse was unable. Participants performed 8–15 repetitions at intensities that went from 4 to 15% BW in weighted vest for lower body, and weight that could be lifted 15 times to a heavier weight that could be lifted 8 times for upper body using free weights.	Exercise intensities were tailored based on body weight and physical limitations.

*FITT* frequency/intensity/time/type, *HR* heart rate, *PT* personal training, *HRR* heart rate reserve, *RPE* rate of perceived exertion, *PA* physical activity, *TTM* transtheoretical model, *SCT* social cognitive theory, *MG* master gardener, *SEM* social ecological model, *URCC* University of Rochester Cancer Center, *W* watts, *RPM* revolution per minute, *QoL* quality of life, *MET* metabolic equivalents, *BW* body weight

### Outcomes

Study feasibility was stated as the primary outcome for seven studies [47, 48, 50, 54, 55, 58, 60], of which six were deemed feasible based on recruitment, retention, adherence, and compliance rates [47, 48, 50, 54, 55, 60]. Though attendance and compliance rates were high, one study was deemed not feasible due to the large number of dropouts owing to the more severe illness of bladder cancer patients [58]. Change in activity or diet behaviour was a primary outcome for two studies [48, 49] and five focused on physical [52, 56–58] or cognitive functioning [54]. Seven found significant improvements in the primary outcome [48–50, 52, 54, 56, 57] while one found no significant group difference [58], though this was a feasibility trial. Further details can be found in Table 3.

**Table 3:** Study results

Source	Outcome(s)	Feasibility results	Primary outcome results	QoL results	Follow-up results	Modifications/changes to intervention
Alibhai 2019 [47]	Feasibility Physical fitness Cost-effectiveness General and prostate-specific QoL: FACT-G plus FACT-P Fatigue: FACT-F	Recruitment rate: 25.4% (59/232 eligible participants) Retention rate at 6 months: 76.3% QoL outcome captur0:e 80% Satisfaction of at least 4 out of 5: 88%	See feasibility	FACT-P HOME group poorer QoL at 6 months than PT difference in Δ = 4.3, (95% CrI − 8.1 to − 0.5, probability of inferiority = 74%) GROUP Δ = − 1.4 (95% CrI − 5.4 to 2.6, probability of inferiority = 21%). FACT-G the change from baseline to 6 months was 2.9 points worse for HOME and 1.7 points worse for GROUP than PT, with the probability of inferiority being 38 and 26%, respectively. Changes in FACT-F were similar between arms.	Not reported	
Bourke 2011 [48]	Feasibility Diet and exercise behaviour Prostate specific QoL; FACT-G and FACT-P Fatigue; FACT-F	Retention 12 weeks intervention = 84% control = 88% 6 months intervention = 60% control = 52% Attendance (for 21/25 men) 360/378 sessions (95%) Compliance in sessions 329/378 (87%) at least 25–30 min recorded in logs.	Total PA behaviour higher in intervention group post intervention Godin LSI points 33.8 vs 17.4 (mean diff Δ = 16.3, 95% CI 8.8–23.8; *p* < .001) and 6 months (25.9 vs. 15.6 Godin LSI points, mean diff Δ = 11.3, 5.0–17.5; *p* = .001) Diet macronutrient intake reductions in total energy intake (mean diff Δ = − 285.5 kcal, − 32.5 to − 484.5; *p* = .005), total fat (mean diff Δ = − 19.8 g, − 7.3 to − 32.3; *p* < .001), saturated fat (mean diff Δ = − 8.6 g, − 3.7 to − 13.5; *p* < .001), and monounsaturated fat intake (mean diff Δ = − 6.6 g, − 2.0 to − 11.2; *p* < .001)	Fatigue (FACT-F) Improvement at 12 weeks in intervention group (mean diff Δ = 5.4, 95% CI = 0.8–10.0; adjusted *p* = .002) FACT-G No significant differences in groups at 12 weeks (*p* = .25) FACT-P No significant differences in groups at 12 weeks (*p* = .21)	6-month follow-up Total PA behaviour higher in intervention group post intervention Godin LSI points 6 months (25.9 vs. 15.6 Godin LSI points, mean diff Δ = 11.3, 5.0–17.5; *p* = .001) Fatigue improvements maintained at 6-month follow-up (mean diff Δ = 3.1, 95% CI = 0.3 to 6.4; adjusted *p* = .006) FACT-G No significant differences in groups at 6 months (*p* = .36) FACT-P No significant differences in groups at 6 months (*p* = .45)	
Demark-Wahnefried 2006 [49]	Diet quality Physical activity behaviour General and cancer specific QoL; FACT-G plus FACT-B or FACT-P	3000 participants identified by cancer registries, 74% had sufficient data to enable contact 688/2010 contactable patients returned consent forms and screeners for 34% response rate Of these, 182 were enrolled and eligible (26%) 168/182 completed all telephone counselling sessions 160/182 completed 12 month follow-up data (cumulative dropout rate of 12.1%)	Behaviour change Improvement in diet quality (between group *p* < .003) for intervention	Enhanced physical functioning though non-significant (between group *p* = .23) Depression scores improved in both groups (between group *p* = .55) FACT-G QoL Both group improved at 6 months (between group *p* = .38)	All scores returned to near baseline levels except QoL which remained at 6 month levels for both groups.	
Demark-Wahnefried 2018 [50]	Feasibility Absence of serious events General QoL; SF-36	Invitation letter mailed = 694 Contactable pool = 624 Enrolled and consented = 46; 24 intervention, 22 WL control Retention rates 22/24 (92%) intervention; 20/22 (91%) WL control	No changes in self-efficacy as it was high to begin with. Increase in intervention arm in social support to garden (*p* = .002) Increased F&V intake among intervention (within group *p* = .02) but not control (between group *p* = .06) Both arms had improvements in physical performance	Perceived stress stable within both arms Reassurance of worth increased in intervention arm whilst decreasing in WLC (between group *p* = .02) More positive results in SF-36 QoL measures for WLC versus intervention group; i.e. pain worsened (*p* = .02) in intervention and physical role (*p* = .01) and overall mental health (*p* = .01) improved in controls	Not reported	
Desbiens 2017 [51]	Fatigue General and breast specific QoL; FACT-G and FACT-B	Not explicitly stated but the challenge of recruitment were a limitation. They were unable to perform two of their intended study objectives.	FACT-F non-significant improvement within both groups; no differences between groups No group difference mean that individual exercise was also potentially effective.	FACT-G non-significant trends towards improvement within both groups; no differences between groups FACT-B non-significant trends towards improvement within both groups; no differences between groups	N/A	Subsequent survey was administered to assess willingness to exercise because accrual was poor
Lai 2017 [52]	Cardiopulmonary intolerance Pulmonary function Cancer specific HRQoL; EORTC QLQ-C30 lung supplement	None explicitly stated	The mean postoperative length of stay (6.9 ± 4.4 vs. 10.7 ± 6.4 days, *p* = .010) and total in-hospital stay (16.0 ± 4.5 vs 19.7 ± 6.5 days, *p* = .012) were significantly reduced in the PR group 6MWT and peak expiratory flow increased significantly in PR group. Potentially due to fewer post pulmonary op complications.	EORTC-QLQC30 & EORTC-LC13_CN no difference was observed between the groups in terms of: global QoL (− 0.5; *P* = .785) physical function (− 0.67; *P* = .691) emotional function (− 2.2; *P* = .206) dyspnoea score (0.37; *P* = .808)	N/A	
Loh 2019 [53]	Anxiety Mood Social and emotional well-being using subscales from FACT-G	N/A	Anxiety within group change for intervention (− 3.51, *p* = .003); between group difference favouring intervention for 75th (− 5.39, *p* = .001) and 95th (− 10.97, *p* < .001) percentiles Mood within group change for intervention (3.08, *p* = .046) and control (4.57, *p* = .002); between group difference favouring intervention for 75th (− 5.04, *p* = .032) and 95th (− 11.12, *p* = .007) percentiles	SWB no significant within group change for intervention or control; between group difference favouring intervention for 5th (3.90, *p* < .001) and 25th (1.39, *p* = .006) percentiles EWB within group change for intervention (1.04, *p* < .001) and control (0.80, *p* = .010); between group difference favouring intervention for 5th (1.82, *p* = .025) percentile	N/A	
Miki 2014 [54]	Feasibility Cognitive function Cancer specific QoL; FACT-G	Deemed feasible due to no dropouts in either group and no adverse events in the intervention group. Highly accepted based on patient reports of how fun it was.	Cognitive function with Frontal Assessment Battery (FAB) found significant time effect (F = 24.39, *p* < 0.001, partial η2 = 0.247) and a significant group effect (F = 9.26, *p* = 0.003, partial η2 = 0.109) Also significant interaction between the two groups on the FAB score (F = 7.88, *p* = 0.006, partial η2 = 0.094)	FACT-G Baseline scores 75.29(15.76) vs. 74.30(14.27) Week 4 scores 77.47(14.01) vs. 75.42(15.42) Interaction *p* = .738; time effect *p* = 0.297; group effect *p* = .612	N/A	
Monga 2007 [55]	Fatigue General and prostate-specific QoL; FACT-G plus FACT-P	None explicitly stated however Nine patients (4 after enrolment, 5 after randomisation) refused to participate, because they wanted to be in the intervention group. Three of the 5 patients who disenrolled after randomisation and initial baseline testing were from the control group	Fatigue with Piper Fatigue scale (higher scores = greater fatigue) Significant between group differences in favour of exercise group for fatigue (change − 4.3 ± 2.1; *t* = − 4.72, *p* ≤ .001)	Within intervention group changes: FACT-G Physical well-being (PWB: change 2.3 ± 1.8; *t* = 4.20, *p* = .002), social well-being (SWB: change 1.5 ± 1.9; *t* = 2.67, *p* = .02) FACT-P (change 7.4 ± 10.4; *t* = 2.36, *p* = .04) Within control group changes SWB (change − 1.7 ± 2.4; *t* = − 2.28, *p* = .05) Between group changes in favour of intervention PWB (change 3.6 ± 2.0; *t* = 4.19, *p* ≤ .001) SWB (change 3.2 ± 2.1; *t* = 3.47, *p* ≤ .002) Functional well-being (FWB: change 4.1 ± 4.2; *t* = 2.24, *p* = .04) FACT-P (change 13.8 ± 10.1; *t* = 3.12, *p* = .006)	N/A	
Morey 2009 [56]	Functional status General QoL; SF-36	558/641 (87%) completed 12 month measures 488/641 (76%) completed 2-year measures.	Change in functional status using physical function subscale of SF-36 (higher score means better function) and Late Life Function and Disability Index (basic and advanced lower extremity function subscales) Control group experienced a mean score change of − 4.84 (95% CI, − 3.04 to − 6.63); more than double that of the intervention group (− 2.15 [95% CI, − 0.36 to − 3.93]); group difference *p* = .03. Significant difference between groups in basic lower extremity function as function changed negligibly in the intervention group (mean, 0.34 [95% CI, − 0.84 to 1.52]), but control group showed a decrease (− 1.89 [95% CI, − 0.70 to − 3.09]; group difference *P* = .005).	Full SF-36 Overall HRQoL decreased in every subscale in the control group. In the intervention group, decreases in subscale scores were of lower magnitude and were sustained for overall health and mental health Overall HrQoL score mean change between groups was 2.71 (95%CI, 0.58 to 4.84); *p* = .03	Not reported	
Park 2012 [57]	Functional physical fitness General QoL; SF-36	No side effects or safety issues arose from exercise programme. 26/33 completed trial and were analysed from exercise group 25/33 completed trial from control group; 2 were excluded from analyses due to missing data	Functional physical fitness Intervention group had greater improvements in fitness (*p* < .001), flexibility (*p* = .027), and balance (*p* = .015).	SF-36 Physical composite score of SF-36 decreased about the same in both groups after surgery (*p* < .001). Physical score recovered to preoperative level in exercise group (*p* < .001) but not in control group (*p* = .225) after 12 weeks. Mental composite score improved after 12 weeks in exercise group (*p* = .017) but not control group (*p* = .773).	N/A	
Porserud 2014 [58]	Feasibility Physical function General QoL; SF-36	Deemed not feasible due to unknown number who were not interested, unknown number not invited to participate, large number of dropouts. 5/9 intervention; 8/9 control completed week 14 measures 4/9 intervention; 6/9 control completed 1 year measures attended 76% of groups exercise sessions and taken daily walks on 87% of the days in the 12 week period	Both groups improved 6MWD	SF-36 Role physical domain in intervention group improved more than control (*p* = .031) after intervention (14 weeks). No other differences were observed at 14 weeks.	No differences remained or were observed at 1-year follow-up.	
Sprod 2015 [59]	Cancer-related fatigue Global side-effect burden; Clinical Symptom Inventory	attendance in sessions averaged 6.2/8 sessions Original study had 410 randomised (206;204) and retained 361 (174;187). Unknown what retention rates were for specific age group of interest in the current study.	Cancer related fatigue (CRF) with Multidimensional Fatigue Symptom Inventory — Short Form (MFSI-SF) Yocas group reported significantly lower CRF than WLC (total score; *p* = .03), physical fatigue (*p* < .01) and mental fatigue (*p* < .01).	Global side effect burden with Clinical Symptom inventory Yocas group had significantly lower level of global side effect burden (*p* < .01)	N/A	
Winters-Stone 2016 [60]	Feasibility General Physical and Mental QoL; Physical and Mental summary scores from SF-36	22% enrolment rate No dropouts in intervention group but 5 couples dropped out of WLC Median attendance to exercise sessions was 78% for PCS, 76% for spouses, and 75% for couples 94% were fully compliant with training	Feasibility primary outcome	Physical and mental summary components of SF-36 No significant group differences among men. Among spouses, mental health increased in intervention while WLC decreased slightly; however, non-significant (*p* = .06). No significant group differences in physical function and vitality subscales of SF-36 in either PCS or spouses.	N/A	

*QoL* quality of life, *PT* personal training, *FACT-G* functional assessment of cancer therapy-general, *FACT-P* FACT-prostate, *FACT-B* FACT-breast, *FACT-F* FACT-fatigue, *SF-36* Short form-36, *EORTC QLQ-C30* European Organisation for Research and Treatment of Cancer QoL Questionnaire 30, *LSI* leisure score index, *F&V* fruits & vegetables, *WLC* wait list control, *SWB* social welling, *PWB* physical well-being, *EWB* emotional well-being, *CI* confidence interval, *6MWD* 6-min walk distance, *CRF* cancer-related fatigue, *HrQoL* health-related quality of life, *PCS* prostate cancer survivors

To measure QoL, five studies used the Medical Outcomes Study’s Short-Form 36 (SF-36) [50, 56–58, 60], five used all or part of the general Functional Assessment of Cancer Therapy (FACT-G) [47, 48, 51, 53, 54] and one used the European Organization for Research and Treatment of Cancer QoL Questionnaire 30 (EORTC-QLQ-30) [52]. Six used cancer-specific measures including the FACT-Prostate [47–49, 55], FACT-Breast [49, 51] and the EORTC Lung Cancer supplement [52]. Six studies assessed symptom specific QoL including fatigue [47, 48, 51, 55, 59], anxiety and mood [53] and side effect burden [59]. Six studies reported significant intervention group improvements [48, 55–59] and six reported no significant group difference in one or more QoL measure [48, 49, 51, 52, 54, 60]. Two studies reported significant negative intervention difference in QoL [47, 50]. There was no difference in effectiveness between studies that reported using theory/psychosocial components to guide the interventions versus those reporting no theory. Three studies using theory reported significant group differences in QoL measures [47, 50, 56]; however, two of these were favouring the non-intervention group [47, 50]. Both theory-based and non-theory-based studies had within group improvements in both quality of life and physical outcomes. Select QoL results are described below; full details can be found in Tables 3 and 4.

**Table 4:** Effectiveness of interventions

Source	Behaviour focus	Theoretical component (yes/no)	Self-tracking (yes/no)	Supervision/counselling component(s)	Level of tailoring	Main outcome effect^a^	QoL effect
Alibhai 2019 [47]	Activity	Minimal – action/coping planning	Yes	1to1 vs F2F Group (G) vs Phone calls (HB)	Minimal based on baseline fitness	Probability of inferiority VO_2_ • GvsPT: 8.2%^b^ • HBvsPT: 26.7%^b^	Probability of inferiority FACT-P • GvsPT: 20.9%^b^ • HBvsPT: 74.4%^b^ FACT-G • GvsPT: 25.6%^b^ • HBvsPT: 37.9%^b^
Bourke 2011 [48]	Nutrition and activity	Minimal – habit formation, autonomy	Yes	F2F education classes	High – feedback and messages	• Godin LSI: between group diff = 16.3, 95%CI (8.8 to 23.8); *p* < .001^c^ • Daily kcal: between group diff = − 258.5, (− 32.5 to − 484.5); *p* = .005^c^	• FACT-P: 5.5 (− 4.2 to 15.3); *p* = .21 • FACT-G: 3.6 (− 3.9 to 11.0); *p* = .25 • FACT-F: 5.4 (0.8–10.0); *p* = .002^c^ (all between group diff)
Demark-Wahnefried 2006 [49]	Nutrition and activity	Yes – SCT, TTM	Yes	Phone	High – feedback and messages	• Diet quality: between group diff + 5.1, *p* = .0026^c^ • PF: between group diff = + 3.6; *p* = .23	• FACT-G: between group diff − 0.3, *p* = .38
Demark-Wahnefried 2018 [50]	Nutrition	Yes – SCT, SEM	No	Home visits & phone	Minimal – plant preferences	• Veg&Fruit per day: between group diff *p* < .06 • Abdominal obesity (cm): between group diff *p* = .05	• SF-36 Physical and Mental summary scores: between-arm diff *p* < .05^b^ • Pain between group diff *p* < .01^b^
Desbiens 2017 [51]	Activity	No	Minimal^d^	None vs F2F	None	• FACT-F^e^: no change either group • BMI^e^: within group diff: G: − 2.3; *p* < .05	• FACT-G^e^: no change either group • FACT-B^e^: no change either group
Lai 2017 [52]	Activity	No	No	F2F	None	• 6MWD: between groups diff + 19.2 m; *p* = .029^c^ • PEF: between groups diff: + 18.0 L/min *p* < .001^c^	• Global QoL: between groups diff: − 0.5; *p* = .785
Loh 2019 [53]	Activity	No	Yes	F2F education	Minimal – based on baseline step count	• STAI: 75th percentile between group diff = − 5.39; *p* = .001^c^; 90th percentile between group diff = − 10.97; *p* < .001^c^ • POMS: 75th percentile between group diff = − 5.04; *p* = .032^c^; 90th percentile between group diff = − 11.12; *p* = .007^c^	• SWB: 5th percentile between group diff = 3.90; *p* < .001^c^; 25th percentile between group diff = 1.39; *p* = .006^c^ • EWB: 5th percentile between group diff = 1.82; *p* = .026^c^
Miki 2014 [54]	Activity	No	No	F2F	None	• FAB: group*time interaction F = 7.88; *p* = .006^c^	• FACT-G: no interaction (*p* = .74) or group effect (*p* = .61)
Monga 2007 [55]	Activity	No	No	F2F	Minimal – based on HR measures	• PFS: between group diff *t* = − 4.72; < .001^c^	• PWB: *t* = 4.19; < .001^c^ • SWB: 3.47; < .002^c^ • EWB: − 0.73; *p* = .48 • FWB: 2.24; *p* = .04^c^ (all between group diff)
Morey 2009 [56]	Nutrition and Activity	Yes – SCT	Yes	Phone	High – feedback and messages	• SF-36 PF: between group diff = 2.69; *p* = .03^c^	• QoL: between group diff = 2.71; *p* = .02^c^
Park 2012 [57]	Activity	No	No	F2F^f^	Minimal – based on HRR	• FPF: between group diff *p* < .001^c^	• Physical QoL: between group diff recovery *p* < .001^c^ • Mental QoL: between group diff recovery *p* = .017^c^
Porserud 2014 [58]	Activity	No	No	F2F Group	Minimal – adaptations for abilities	• 6MWD improved: between group diff *p* = .013^c^	• SF-36 role physical improved between group diff *p* = .031^c^
Sprod 2015 [59]	Activity	No	No	F2F Group	None	• CRF: between group diff = − 5.5; *p* = .03^c^	• CSI: between group diff = − 4.51; *p* = .009^c^
Winters-Stone 2016 [60]	Activity	Minimal – social support	No	F2F Group	Minimal – based on BW and limitations	• Bench press (kg): between group diff = 0.62; *p* < .01^c^ • SR weekly MET between group diff = 303.60; *p* < .01^c^	• Physical QoL: between group diff *p* = .99 • Mental QoL: between group diff *p* = .39

^a^If feasibility was primary outcome, candidate primary outcome presented

^b^Favouring control group

^c^Favouring intervention group

^d^Number of times completed video workout

^e^No between group analyses completed

^f^Unknown whether individual or group-based

*PA* physical activity, *TTM* transtheoretical model, *SCT* social cognitive theory, *SEM* social ecological model, *F2F* face to face, *G* group, *PT* personal training, *HB* home-based, *HR* heart rate, *HRR* heart rate reserve, *BW* body weight, *V02* maximum rate of oxygen consumption, *LSI* leisure score index, *kcal* kilocalorie, *cm* centimetre, *BMI* body mass index, *6MWD* 6-min walk distance, *PEF* peak expiratory flow, *PF* physical function, *FPF* functional physical fitness, *kg* kilogramme, *FACT-G* functional assessment of cancer therapy-general, *FACT-P* FACT-prostate, *FACT-B* FACT-breast, *FACT-F* FACT-fatigue, *PFS* Piper Fatigue Scale, *POMS* Profile of Mood States, *STAI* State Trait Anxiety Inventory, *FAB* functional assessment battery, *SWB* social well-being, *PWB* physical well-being, *EWB* emotional well-being, *PFS SF-36* short form-36, *CRF* cancer-related fatigue, *CSI* clinical symptom inventory, *QoL* quality of life, *CI* confidence interval, *SR* self-reported, *MET* metabolic equivalent

### Quality of life

Participants in a home- or group-based activity programme reported poorer general and cancer-specific QoL than those in a 1:1 personal training group [47]. Participants that received a home-based personalized activity and nutrition intervention reported similar improvements in general QoL to an attention control group receiving general information at study end which was maintained at follow-up [49]. A wait-list control group showed more positive improvement in scores for pain, physical role and overall mental health compared to those receiving a gardening intervention [50]. Both the home-based and group session-based participants in an exercise intervention for women diagnosed with breast cancer improved their overall and breast cancer-specific QoL [51]. No differences were observed between groups on global or lung cancer-specific QoL in those participating in a prehabilitation intervention versus usual care [52]. In a group of mixed cancer patients undergoing chemotherapy, between group differences favouring the intervention were noted among those having poorer social and emotional well-being at baseline measures compared to the wait-list control [53]. No between-group or within-group differences were found for QoL in speed feedback therapy group versus usual care [54]. Those receiving a supervised activity programme had significant between-group improvements in overall and prostate cancer-specific QoL compared to those receiving usual care [55]. While both groups in a home-based activity and nutrition intervention versus wait-list control had declines in overall QoL throughout the study period, the intervention group had significantly smaller declines than the control group [56]. Physical and mental composite scores of the SF-36 returned to preoperative levels in participants in a functional exercise intervention when compared to those in a usual care group [57]. In an exercise study among people with bladder cancer, only the role-physical domain scores improved significantly in the intervention group compared to usual care; all other scores had no differences [58]. No differences were found between exercise and control groups in men with prostate cancer, but among spouses also participating, there was a non-significant increase in partners’ mental health scores [60].

### Fatigue and other side effects

Men starting or currently on androgen deprivation therapy (ADT) reported a similar change in fatigue among three groups receiving an activity programme [47]. Participants that received a combination of supervised and home-based activity reported more improvement in fatigue than control groups after the study period which was maintained at follow-up [48]. An activity and nutrition intervention found improvement in depression scores in both the tailored versus non-tailored groups [49].

In a study comparing the same programme either home or group based, there were non-significant improvements in fatigue in both groups [51]. Participants receiving a low- to moderate-intensity home-based activity programme, with poorer anxiety and mood at baseline, had significant improvements compared to the control group [53]. Men with prostate cancer participating in aerobic exercise before radiotherapy reported significantly better fatigue scores than those in usual care [55]. Those participating in a yoga intervention reported significantly lower cancer-related fatigue and global side effect burden than the wait-list control group [59].

## Discussion

This review describes the current literature around the nature of PA and nutrition interventions for older adults with cancer. Our initial inclusion age criterion was “aged 70 or older”; however, we had to amend this to 60 or older as we retrieved no studies that met all criteria illustrating the relative paucity of literature relating to older adults. Most available research has targeted relatively young people living with and beyond cancer, limiting the relevance of subsequent clinical guidance to older adults [43, 69–71].

We found 14 RCTs relevant to our question; most were feasibility/pilot trials, but 6 were evaluation phase studies. Effects on QoL outcomes were unsurprisingly mixed given that most were not designed to test effectiveness; however, the evaluation phase trials showed positive trends in QoL related to lifestyle interventions [49, 51–53, 55, 56]. Trials were globally representative across North America, the Far East and Europe and across all healthcare settings. Most studies were in people with prostate cancer and few included people with advanced disease; even the lung cancer prehabilitation trial included people eligible for radical surgery. Three interventions included both nutrition and PA components, with the vast majority of trials investigating a PA intervention only. Overall, our main findings are that older adults should be considered as a different population, tailoring of interventions increases relevance to the patient and a holistic approach with attention to behavioural self-management strategies with at least some personal contact with a therapist or health professional seems to be necessary.

Compared with the cancer adult population as a whole, older adults have more comorbidities, are at more risk for falls and frailty and current guidelines for behaviour change may not be relevant. However, from studies of pulmonary rehabilitation in non-malignant lung disease, older adults gain as much benefit as younger patients from such interventions although completion rates are lower; those with frailty being twice as likely not to complete [24, 72, 73]. Therefore, tailoring interventions is important. In the behaviour change field, studies that tailor education or interventions to individual participants are more likely to result in meaningful behaviour change [74, 75]. Tailored messages are more personally relevant and are more likely to be read, understood, recalled, higher rated and seen as credible than generalized messages [74, 76]. In this review, studies that tailored programmes to participants’ individual capacity and preference were more likely to lead to change behaviour and in QoL measures [48–50, 57]. The more tailored a programme is, the more relevant it will be and is more likely to result in behaviour change [76]. A recent systematic review highlighted the lack of behaviour change technique (BCT) use among thoracic cancer interventions [77]. Though we found mixed results of the effectiveness of theory-based interventions in our review, lifestyle behaviour change programmes that use appropriate BCTs to guide interventions are generally more effective [78].

The subjective nature of QoL may be different for older adults versus younger groups. Values and goals may shift for those in older age and the approach taken for lifestyle behaviour change must reflect this. Research in older adults in the general population highlights the need for more focus on functional fitness and mental well-being to remain independent [44]. The goal in this population is to live as well as possible, for as long as possible. This is reflected in the older adult cancer population where the goal or desired outcome is often functional, not fitness [44]. In this review, the majority of studies found improvements in physical function measures. One study demonstrated fewer days post-surgical recovery and a shorter hospital stay in the intervention group [52], while another study, delivering yoga found reductions in cancer-related fatigue, physical and mental fatigue, and a lower side effect burden [59]. Programmes that are more holistic in nature, focusing on both physical and mental wellness, may be most appropriate, seen as more relevant, and thereby garnering greater engagement.

Interventions with positive QoL outcomes had some form of supervised instruction or training with qualified professionals. Studies that had at least one face-to-face session were more likely to have greater positive changes in QoL measures than those that were home-based only [47, 53, 55, 57, 59]. In some studies, this was only an introductory session. While supervised activity sessions tend to have higher adherence and satisfaction, they are more expensive and resource intensive. However, studies that included telephone professional support also found positive results [49, 56]. The amount of supervision needed to make a lasting difference, or the appropriate “dose” for instigating behaviour change is unknown, and a recommended avenue of study [79].

Most of the study participants were breast or prostate cancer patients during or having completed treatment. While providing important perspectives, this reduces the generalizability of recommendations outside of these groups. In particular, only two studies included people with more severe disease: one with lung cancer [57] and one in bladder cancer [58]. The bladder cancer intervention was deemed not feasible, despite the positive outcomes in the intervention group, due to the low recruitment rate and number of drop-outs highlighting the difficulty in delivering programmes to sicker patients with more comorbidities [80–82]. Future research should focus on ways to ensure the most appropriate programmes for these populations by development work with their target populations.

The study among lung cancer patients was also the only prehabilitation study, aimed at providing a programme designed to improve functional outcomes that would therefore reduce post-surgical complications. Participants will have been assessed as fit enough for surgery, thereby not informing clinical practice for most older adults with lung cancer. Further, while potentially very beneficial, prehabilitation studies are difficult to implement given the short time-frames necessary prior to radical cancer treatments [79, 83]. Future research needs to investigate how to deliver interventions in as short a time as possible to have meaningful impact on patient-centred outcomes among those eligible for surgery.

Most studies focused on PA behaviour, highlighting the lack of nutrition interventions in older adults living with and beyond cancer despite nutritional status being a predictor for poor clinical outcomes [84]. Among the included studies, only four had a nutrition component [48–50, 56]. The majority of advice was general and focused on comparing current nutritional intake to national guidelines [48, 50, 56]. Only one study that used a nutrition intervention provided more detailed and tailored advice [49]. Diet patterns have been shown to influence QoL in older adult populations [85] but little research is available that tests the differential effects of tailored PA, diet or a combination of both. More research is necessary, particularly randomized controlled trials, to determine the presence and strength of this link among the older adult LWBC population.

### Limitations and strengths

Strengths include a broad search method and the use of independent researchers. However, as with any review, important papers may have been missed. As with much of cancer research, most patient groups in this review were either breast or prostate cancer reducing the generalizability of the findings. Over half of the articles included were identified as feasibility studies. Though the majority of studies indicated the interventions were feasible, they were underpowered for effectiveness. Finally, few studies indicated using any theoretical base or specific BCTs. Future research should incorporate appropriate techniques to assist self-management and help encourage higher completion rates for older adults, especially those with frailty and sarcopenia, learning from research in other conditions as relevant [72].

### Implications for research

This work identified key gaps in the evidence supporting rehabilitation-based programmes for older adults with cancer, and a paucity of work including nutrition interventions alongside those aiming to improve PA. Development of acceptable and relevant interventions, flexible across the cancer continuum and cancer type and stage are needed. One size is unlikely to fit all. Future research should be underpinned by behaviour change theory and include studies to explore how best to support attendance and completion by those with frailty and sarcopenia. There is likely to be overlap with research in other areas of older adults’ health and rehabilitation but although there is interest in generic rehabilitation programmes, there is little evidence to date to confirm benefits in people with cancer, or in older adults [86, 87].

## Conclusions

This review identified very little research that focused on older adults specifically despite the growing proportion of this group. Few studies included a nutritional component. Findings useful to inform the design of activity/nutrition programmes include candidate intervention components, the need to use a holistic and tailored approach with functional goals and some personal professional contact. The tailoring must take into account the older person’s personal goals and be flexible along the cancer continuum depending on current treatment plans. Learning from general older adult populations as well as rehabilitation literature in other disease groups, e.g. chronic obstructive pulmonary disease, will help advance this research.

## Electronic supplementary material

ESM 1 (DOCX 28 kb)
