# Supplementary material for: Physical activity and nutrition interventions for older adults with cancer: a systematic review
Source: J Cancer Surviv. 2020 Apr 24;14(5):689–711. doi: 10.1007/s11764-020-00883-x (PMC7473955; doi:10.1007/s11764-020-00883-x)
Supplement: Supplementary file 1 — (DOCX 28 kb) [file 11764_2020_883_MOESM1_ESM.docx]

**Title:** Physical activity and nutrition interventions for older adults with cancer: a systematic review.

**Submitted to**: Journal of Cancer Survivorship.

**Authors**: Cynthia C. Forbes, Sarah L. Greenley, Flavia Swan, Michael Lind, Miriam J. Johnson

**Corresponding author**: Cynthia C Forbes, University of Hull, [cindy.forbes@hyms.ac.uk](mailto:cindy.forbes@hyms.ac.uk)

**Supplemental materials**

**Original Medline Search strategy (others adapted from this)**

1. exp AGED/
2. exp CACHEXIA/
3. (elder* or frail* or old* or geriatric).mp. [mp=title, abstract, original title, name of substance word, subject heading word, floating sub-heading word, keyword heading word, protocol supplementary concept word, rare disease supplementary concept word, unique identifier, synonyms]
4. 1 or 2 or 3
5. exp NEOPLASMS/
6. cancer*.mp. [mp=title, abstract, original title, name of substance word, subject heading word, floating sub-heading word, keyword heading word, protocol supplementary concept word, rare disease supplementary concept word, unique identifier, synonyms]
7. 5 or 6
8. exp REHABILITATION/
9. exp EXERCISE/
10. exp Physical Fitness/
11. (physical adj1 (activit* or training or fitness or conditioning)).mp. [mp=title, abstract, original title, name of substance word, subject heading word, floating sub-heading word, keyword heading word, protocol supplementary concept word, rare disease supplementary concept word, unique identifier, synonyms]
12. (musc* adj1 (strength or endurance or conditioning)).mp. [mp=title, abstract, original title, name of substance word, subject heading word, floating sub-heading word, keyword heading word, protocol supplementary concept word, rare disease supplementary concept word, unique identifier, synonyms]
13. exp DIET/
14. (diet* or nutrition*).mp. [mp=title, abstract, original title, name of substance word, subject heading word, floating sub-heading word, keyword heading word, protocol supplementary concept word, rare disease supplementary concept word, unique identifier, synonyms]
15. ((diet* or nutrition* or food) adj1 supplement*).mp. [mp=title, abstract, original title, name of substance word, subject heading word, floating sub-heading word, keyword heading word, protocol supplementary concept word, rare disease supplementary concept word, unique identifier, synonyms]
16. 8 or 9 or 10 or 11 or 12 or 13 or 14 or 15
17. exp "Quality of Life"/
18. exp Health Status/
19. ("quality of life" or wellbeing or well-being or well being or health status).mp. [mp=title, abstract, original title, name of substance word, subject heading word, floating sub-heading word, keyword heading word, protocol supplementary concept word, rare disease supplementary concept word, unique identifier, synonyms]
20. hrqol.mp. [mp=title, abstract, original title, name of substance word, subject heading word, floating sub-heading word, keyword heading word, protocol supplementary concept word, rare disease supplementary concept word, unique identifier, synonyms]

21. 17 or 18 or 19 or 20

22. 4 and 7 and 16 and 21

Update Medline Search Strategy (incorporating Cochrane Highly Sensitive Search Strategy for identifying randomized trials in MEDLINE: sensitivity- and precision-maximizing version (2008 revision); Ovid format)

| 1. exp AGED/ |  |
| --- | --- |
| 2. exp CACHEXIA/ |  |
| 3. (elder* or frail* or old* or geriatric).mp. |  |
| 4. 1 or 2 or 3 [ older people concept ] |  |
| 5. exp NEOPLASMS/ |  |
| 6. cancer*.mp. |  |
| 7. 5 or 6 [ cancer concept ] |  |
| 8. exp REHABILITATION/ |  |
| 9. exp EXERCISE/ |  |
| 10. exp Physical Fitness/ |  |
| 11. (physical adj1 (activit* or training or fitness or conditioning)).mp. |  |
| 12. (musc* adj1 (strength or endurance or conditioning)).mp. |  |
| 13. exp DIET/ |  |
| 14. (diet* or nutrition*).mp. |  |
| 15. ((diet* or nutrition* or food) adj1 supplement*).mp. |  |
| 16. 8 or 9 or 10 or 11 or 12 or 13 or 14 or 15 [ diet and exercise intervention ] |  |
| 17. exp "Quality of Life"/ |  |
| 18. exp Health Status/ |  |
| 19. ("quality of life" or wellbeing or well-being or well being or health status).mp. |  |
| 20. hrqol.mp. |  |
| 21. 17 or 18 or 19 or 20 [ quality of life outcome ] |  |
| 22. 4 and 7 and 16 and 21 [ Original MEDLINE search ] |  |
| 23. randomized controlled trial.pt. |  |
| 24. controlled clinical trial.pt. |  |
| 25. randomized.ab. |  |
| 26. placebo.ab. |  |
| 27. clinical trials as topic.sh. |  |
| 28. randomly.ab. |  |
| 29. trial.ti. |  |
| 30. 23 or 24 or 25 or 26 or 27 or 28 or 29 |  |
| 31. exp animals/ not humans.sh. |  |
| 32. 30 not 31 [ Cochrane balanced 2008 RCT filter ] |  |
| 33. 22 and 32 |  |
| 34. limit 33 to ed=20180717-20190531 |  |
| 35. limit 33 to yr="2018 -Current" |  |
| 36. 34 or 35 |  |
